# Evaluating the Learning Curve for Percutaneous Nephrolithotomy under Total Ultrasound Guidance

**DOI:** 10.1371/journal.pone.0132986

**Published:** 2015-08-13

**Authors:** Yan Song, YaNan Ma, YongSheng Song, Xiang Fei

**Affiliations:** 1 Urology Division, Sheng Jing Hospital, China Medical University, Shenyang, Liaoning Province, China; 2 Department of Biostatistics and Epidemiology, School of Public Health, China Medical University, Shenyang, Liaoning Province, China; Cardiff University, UNITED KINGDOM

## Abstract

**Objectives:**

To investigate the learning curve of percutaneous nephrolithotomy under total ultrasound guidance.

**Methods:**

One hundred and twenty consecutive PCNL operations under total ultrasound guidance performed by a novice surgeon in a tertiary referral center were studied. Operations were analyzed in cohorts of 15 to determine when a plateau was reached for the variables such as operation duration, ultrasound screening time, tract dilation time, stone-free rate and complication rate. Comparison was made with the results of a surgeon who had performed more than 1000 PCNLs. Fluoroscopy was not used at all during procedure.

**Results:**

The mean operation time dropped from 82.5 min for the first 15 patients to a mean of 64.7 min for cases 46 through 60(P = 0.047). The ultrasound screening time was a peak of 6.4 min in the first 15 cases, whereas it dropped to a mean of 3.9 min for cases 46 through 60(P = 0.01). The tract dilation time dropped from 4.9 min for the first 15 patients to a mean of 3.8 min for cases 46 through 60(P = 0.036). The senior surgeon had a mean operating time, screening time and tract dilation time equivalent to those of the novice surgeon after 60 cases. There was no significant difference in stone free rate and complication rate.

**Conclusions:**

The competence of ultrasound guided PCNL is reached after 60 cases with good stone free rate and without major complications.

## Introduction

Since its introduction in 1976, percutaneous nephrolithotomy (PCNL) has been considered the preferred approach to large stones, stones in the lower pole, or stones that are resistant to less invasive technology[1]. In our opinion, achieving percutaneous access to the kidney is the procedure’s most important step, and it has an enormous influence on the success and the complication rates associated with the intervention. The punctures are usually performed under fluoroscopic guidance, which exposes the patients and the surgical teams to radiation, and the cumulative effects of the radiation increase the risk to physicians[2]. It has been reported that a novice surgeon can be exposed to radiation doses that are as high as1440 cGy/cm^2^ while undertaking their first procedures; hence, the exposure to radiation is not insignificant[3]. Moreover, the contrast material may conceal the opacity of the stone and this may cause confusion when extravasation occurs[4].

Ultrasonography eliminates the hazards associated with exposure to radiation, and it can be used reliably to locate renal stones, especially non-opaque stones that are not visible using fluoroscopy. Furthermore, neighboring organs such as the bowel, liver, spleen, and lung, can be identified easily and their injury can be avoided during puncture[5].This technique helps the novice surgeon to develop the percutaneous access method, thereby contributing to low complication rates, especially while learning the technique.

To correctly prepare a training program for PCNL, the technique’s learning curve should be determined. A ‘‘learning curve” is defined as a graph that represents progress in the mastery of a skill against the time required for such mastery to be achieved. The point at which no further improvement is observed has been proposed as the point when competence is reached[6]. In urology, interest in characterizing learning curves is currently limited to cancer surgery[7]and robotic laparoscopic procedures[8]. So far, only a handful of studies have been published that have aimed to evaluate the learning curve for PCNL under fluoroscopic guidance, and it has been reported that surgical competence is obtained after the procedure has been performed on 60 patients[9,10].Ultrasound (US)-guided PCNL is practiced in many regions, but no studies have been undertaken to investigate the learning curve for this procedure.

In the present study, we aimed to determine the learning curve associated with a novice surgeon performing solo PCNL completely under US guidance, and to define the number of procedures required to attain competence using specified parameters.

## Patients and Methods

Informed consent have been obtained from the participants before operation. The ethics committee of Sheng Jing Hospital approved this retrospective study; and patient records/information was anonymized and de-identified prior to analysis.

This study was carried out at Sheng Jing Hospital, which has a busy tertiary referral center for complex stone disease, where an average of three PCNL operations occur each working day. The hospital receives more than 1000 referrals per year and it treats patients from northeast China. All of the PCNL operations are performed under US guidance, and fluoroscopic guidance is never used.

Prophylactic antibiotics were administered to all of the patients, and patients who had infections were treated according to their antibiogram results. Patients with ectopic kidneys or urosepsis were excluded from the study. Data about the patients’ ages, sexes, stone types, stone burdens, degrees of hydronephrosis, treatment, and their urolithiasis histories were recorded. For patients for whom PCNL was planned, non-contrast abdominal computed tomography was performed to clarify the sizes and locations of the calculi and to determine their hydronephrosis grades before surgery.

A total of 120 consecutive patients with renal stones were evaluated during an 18-month period. The patients were divided into eight groups according to the time frames in which they underwent PCNL during the 18-month period, and each group comprised 15 subjects. All of the patients had undergone PCNL undertaken by one surgeon who had trained for a fellowship in endourology for 1 year and who had no prior experience of performing solo PCNL. However, the surgeon had experience in performing other endourology procedures, including ureteroscopy, transurethral lithotripsy, and percutaneous US-guided nephrostomy, and he had undergone US training for 2 weeks. After a 1-month observation period that comprised approximately 20 cases who underwent PCNL procedures and scrubbing with an experienced endourologist during 5 operations as the first assistant, the fellow was allowed to perform solo PCNL. The novice surgeon’s results were compared with those from a random cohort of 15 consecutive PCNL procedures performed by the department’s senior surgeon during the study period, which formed a ninth group. The senior surgeon had more than 5 years of experience in percutaneous surgery, and had performed more than 1000 PCNL procedures.

After anesthesia, each patient was initially placed in the lithotomy position, and a ureteral catheter was advanced to the kidney and was secured using a Foley catheter. Subsequently, the patient was turned to the prone position. Gaining entry to the desired calyx was defined as successful access to the collecting system. Ultrasonography was performed to detect the presence of organs in the path of the puncture to avoid organ injury. In the absence of hydronephrosis, saline was infused through the ureteral catheter to ensure the ballooning of the pelvicalceal system (PCS). Mild hydronephrosis was sufficient to access the targeted calyx. A colored-Doppler US system with a 3.5-MHz transducer was used in this study. An 18-gauge coaxial needle was introduced into the most convex point of the target calyx under US guidance, which was undertaken either freehand or using a needle-guidance system fixed to the US probe. A US-guided puncture through the cup of the target calyx was used to traverse the minimum amount of cortical tissue and to establish the shortest straight tract between the skin and the calyx, while avoiding visceral injury. The obturator was removed, and a 5-mL syringe was attached to the needle. With steady suction on the syringe and the recovery of the injected fluid from the ureteral catheter, access to the collecting system was confirmed. After the removal of the stylet, a J-tipped 0.038-in guidewire was inserted, and the length of the needle from the skin to the PCS was measured to ensure that the length of the dilator was appropriate.

A two-step method was used to establish the working channel as we have described previously[11]. First, after a 1.0-cm skin incision was made, the dilation of the percutaneous tract was serially performed over the guidewire using a 16-Fr Amplatz teflon fascial dilator, and a peel-away sheath was inserted. Subsequently, a 9.8-Fr semi-rigid ureteroscope was inserted to inspect the position of the working channel under direct vision and to make minor adjustments, if necessary. For example, if the tip of the peel-away sheath was on the edge of the target calyx, minor adjustments could be made to ensure that the sheath was placed correctly within the collecting system by moving the tip of the sheath to the center of the calyx under direct vision. Second, the channel was further dilated using a coaxial telescoping Alken dilator to establish a 24-Fr working channel over the guide wire. After dilation, a 24-Fr Alken sheath was positioned as the percutaneous access port and a 20.8-Fr rigid nephroscope was applied. Subsequently, conventional calculus disintegration was performed. On the first postoperative day, blood counts were performed to assess changes in the patients’ hematocrit levels, and the Foley catheters were removed, if hematuria was not evident. The nephrostomy tubes were removed 48 h after the operation. Kidney ureter bladder radiography was routinely performed for residual stones 48 h after surgery, and shock-wave lithotripsy or second-look nephroscopies were considered as auxiliary treatments when indicated.

The patients’ demographic information, stone characteristics, and their intra- and postoperative data, including the complications associated with surgery, were evaluated and compared among the groups. Surgical complications were classified using the modified Clavien-Dindo classification of surgical complications[12]. The operative time was defined as the time from the insertion of the cystoscope for the placement of the ureteral occlusion catheter until the access tract was sealed. The US screening times and the tract dilation times were also recorded. A stone-free status was defined as the absence of visible fragments. Clinically insignificant residual fragments (CIRF) were defined as those that were <4 mm in diameter, non-obstructive, and asymptomatic.

The data were statistically analyzed using the Statistical Package for the Social Sciences software version 16.0. The categorical data were compared using the chi-square test. The data are expressed as the means(standard deviations [SD]). A value of *P* <0.05 was considered statistically significant.

## Results

The patients’ demographic data are presented in Table 1.There were no statistically significant differences among the patient groups with respect to age, sex, stone type, and laterality. The mean stone size was 553.73±203.95mm^2^ in the study group. Although the stone sizes seemed to increase in the groups that were operated on at later times as the surgeon gained more experience, there were no significant differences among the groups with respect to stone size(*P* = 0.13).

**Table 1:** Demographic characteristics data of patients according to patients’ group.

	Group 1 (1–15)	Group 2(16–30)	Group 3(31–45)	Group 4(46–60)	Group 5(61–75)	Group 6(76–90)	Group 7(91–105)	Group 8(106–120)	Group 9(Senior surgeon)	p
**Age**	49.20± 5.71	49.73± 9.85	47.87± 4.92	46.60± 6.43	51.20± 8.11	52.33± 7.51	48.67± 6.98	49.07± 5.43	47.13± 3.86	0.763
**Sex** (male/ female)	10/5	9/6	8/7	10/5	8/7	9/6	7/8	10/5	9/6	0.964
**Side** (left/right)	9/6	8/7	7/8	7/8	9/6	7/8	7/8	7/8	6/9	0.983
**Stone type**										0.992
Single (N,%)	13(86.6%)	12(80%)	11(73.3%)	12(80%)	11(73.3%)	12(80%)	11(73.3%)	10(66.7%)	12(80%)	
Mulitple /staghorn (N,%)	2(13.3%)	3(20%)	4(26.7%)	3(20%)	4(26.7%)	3(20%)	4(26.7%)	5(33.3%)	3(20%)	
**Stone area (mm2)**	441.29± 102.99	491.27± 122.19	553.5± 228.18	570.8± 203.07	583.4± 173.16	584.4± 227.71	578.2± 226.48	597.67± 335.29	572.20± 181.52	0.13

Data are presented as mean (SD) except sex, side and stone type which are presented as n (%).

**Fig 1:**
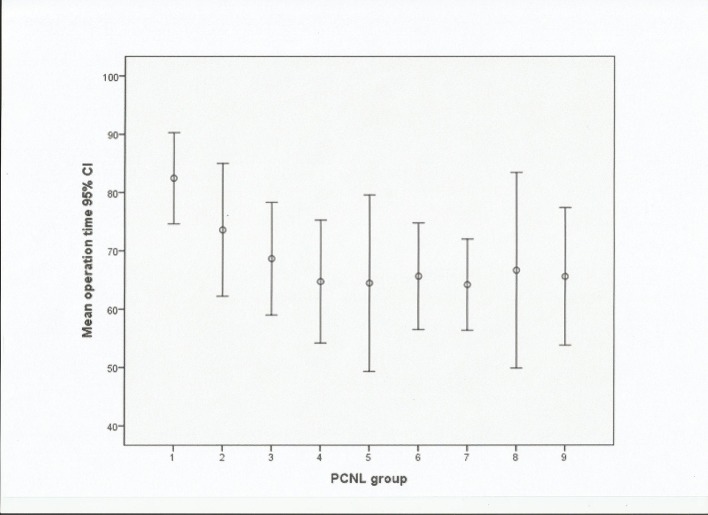
Mean operation time in minutes with 95% confidence interval (CI).

The mean operative times gradually decreased (Fig 1). They ranged from a mean operative time of 82.47±17.22min for the first 15 PCNL procedures to a mean operative time of 64.73±14.21min for case numbers 46–60. The decrease in the mean operative time was statistically significant for the first 60 cases (*P*< 0.05). Then,few fluctuations were observed with respect to the mean operative times in the subsequent patient groups, indicating that a plateau had been reached (*P* = 0.998). The senior surgeon had a mean operative time of 65.60±15.38min, which was similar to that achieved by the novice surgeon after 60 operations.

**Fig 2:**
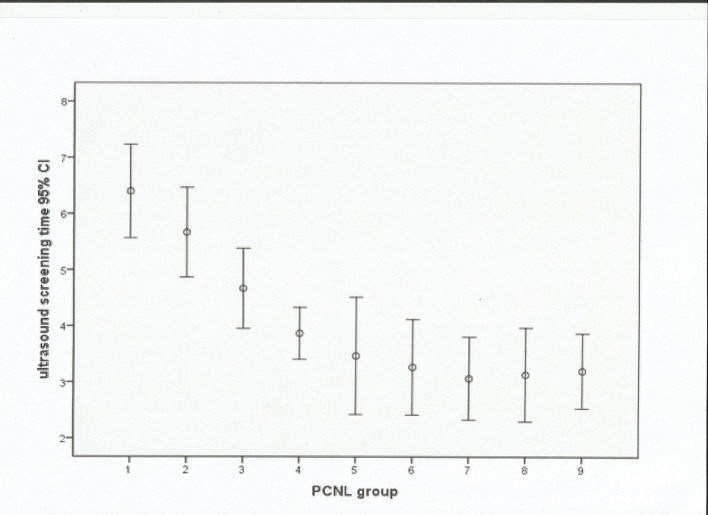
Mean ultrasound screening time in minutes with 95% confidence interval (CI).

The US screening time also reduced as experience in the procedure gradually increased (Fig 2). The mean screening time was 6.40±1.22min for the first 15 cases,and this decreased significantly to a mean screening time of 3.9±0.61min when the sixtieth procedure had been completed(*P*<0.001).This screening time was maintained for case numbers 61–120. The senior surgeon’s mean screening time was 3.2±1.93 min, which was similar to that achieved by the novice surgeon after 60 procedures.

**Fig 3:**
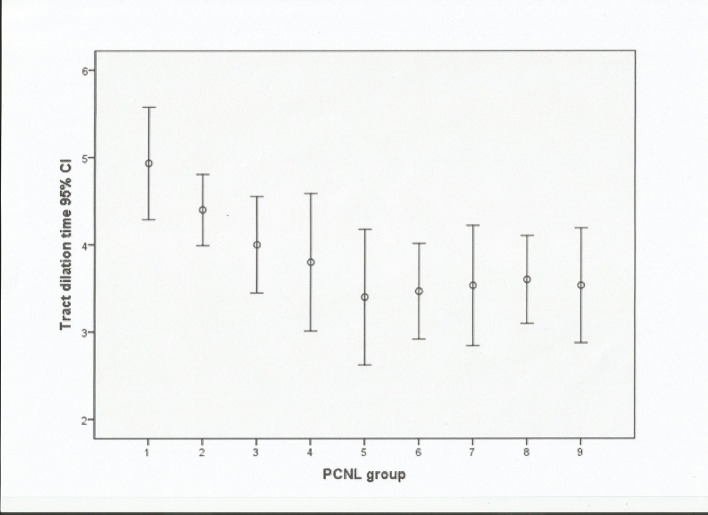
Mean tract dilation time in minutes with 95% confidence interval (CI).

The tract dilation time also decreased (Fig 3).The mean tract dilation time was 4.93±0.89 min for the first 15 PCNL procedures and this decreased to a mean tract dilation time of 3.80±1.15 min for cases 45–60. The decrease in the mean tract dilation time was statistically significant for the first 60 cases (*P*< 0.05),and no further reductions in the tract dilation time occurred. These changes mirrored those that occurred in relation to the screening and operative times.The senior surgeon’s mean tract dilation time was similar to that of the novice surgeon after 60 procedures had been undertaken.

To provide an internal control, we examined the routes of access, the estimated blood losses, the stone-free rates, the durations of the hospital stays, and the complication rates among the PCNL groups.

The numbers for each access route were as follows: lower pole access (*n* = 33), middle pole access (*n* = 80), upper pole access (*n* = 35), and multiple access routes (*n* = 15) (Table 2). There were no significant differences among the groups regarding the access routes and the numbers of access routes(*P* = 0.952).

**Table 2:** Intraoperative and Postoperative data according to patients’ group.

	Group 1 (1–15)	Group 2(16–30)	Group 3 (31–45)	Group 4 (46–60)	Group 5(61–75)	Group 6(76–90)	Group 7(91–105)	Group 8(106–120)	Group 9(Senior surgeon)	P
**Multiple access(N,%)**	1(6.7%)	1(6.7%)	3(20%)	2(13.3%)	2(13.3%)	1(6.7%)	1(6.7%)	2(13.3%)	2(13.3%)	0.952
**Hb drop(g/dl)**	2.53±1.91	4.20±2.11	3.53±1.77	2.73±1.74	2.53±2.19	2.47±1.49	2.60±1.77	2.33±1.78	2.53±1.91	0.071
**Stone free rate (N, %)**	9(60%)	10(66.7%)	12(80.0%)	10(66.7%)	11(73.3%)	12(80.0%)	12(80.0%)	14(93.3%)	12(80.0%)	0.714
**CIRF rate**	4(26.7%)	3(20.0%)	2(13.3%)	3(20%)	2(13.3%)	3(20.0%)	2(13.3%)	0	3(20.0%)	0.852
**Hospital stay(day)**	3.5±0.98	4.1±0.96	3.4±0.95	3.67±0.8	3.60±0.88	3.33±0.62	3.67±0.76	3.73±0.78	3.53±0.98	0.196

Data are presented as mean (SD) except access number, stone free rate and CIRF rate which are presented as n (%).CIRF clinical insignificant residue fragment.

The mean estimated blood losses, which were determined by declines in the hemoglobin levels, ranged from 4.20±2.11 g/dL to 2.33±1.78 g/dL among the PCNL groups, and although there was tendency for lower blood losses among the groups that had undergone operations at later times, the difference was not significant (*P* = 0.071).

The overall stone-free rate was 75% (range 60.0%–93.3%) for all of the groups (Table 2). The stone-free rate gradually increased as the surgeon gained experience, but there were no statistical differences among the groups regarding the stone-free rate (*P* = 0.714) and the CIRF rate (*P* = 0.852).

Complication rates decreased slightly as the novice surgeon gained experience, but the decline in complication rates did not reach statistical significance (*P* = 0.497). There were no major complications according to the modified Clavien-Dindo classification of surgical complications (Table 3).

**Table 3:** Complications of percutaneous nephrolithotomy classified according the modified Clavien system.

	Group 1(1–15)	Group 2(16–30)	Group 3(31–45)	Group 4(46–60)	Group 5(61–75)	Group 6(76–90)	Group 7(91–105)	Group 8(106–120)	Group 9(Senior surgeon)	p
**Grade 1**										
Nephrostomy tube displace-ment	-	1	-	-	-	-	-	-	-	-
Transient fever< 38°C	1	1	1	1	1	1	-	1	1	-
**Grade 2**										
bleeding requiring transfusion	1	2	-	1	-	-	-	1	-	-
Nonseptic infections requiring additional antibiotics	1		1	-	-	-	-	-	-	-
**Grade 3**	-	-	-	-	-	-	-	-	-	-
**Grade 4**	-	-	-	-	-	-	-	-	-	-
**Grade 5**	-	-	-	-	-	-	-	-	-	-
**Overall (N, %)**	3(20%)	4(26.7%)	2(13.3%)	2(13.3%)	1(6.7%)	1(6.7%)	0	2(13.3%)	1(6.7%)	0.497

Data are presented as n (%).

## Discussion

PCNL has replaced open surgery in the treatment of renal stone disease, which motivates surgeons to learn this procedure. However, it is an invasive procedure and it is associated with a risk of complications. The renal pelvis perforation, hydrothorax, and significant bleeding rates have been reported at 3.4%, 1.8%, and 7.8%, respectively, and blood transfusion is sometimes required[13,14]. Severe complications, including colon and spleen injuries have also been reported[15]. Watterson reported that only 11% of urologists obtained access themselves, and that many relied on radiologists for this step of the procedure, but the stone clearance rates were higher and the complication rates were lower when access was obtained by a urologist[16]. Hence, it is very important for surgeons to know how to obtain access themselves, which certainly requires longer training times. However, the length of the training period is not an appropriate criterion on which to judge competence, because the numbers of operations performed vary greatly among teaching centers[17]. It is important to determine the number of operations required to achieve competence in PCNL and the factors that might impact upon surgical competence in PCNL procedures.

To better define the learning curve for US-guided PCNL, we evaluated a number of parameters, including the operative and US times, the stone-free rates, and the complication rates. Often, the most time-consuming and challenging parts of PCNL are the initial puncture and tract dilation. A significant reduction in the US screening time is likely to reflect a growing understanding of the surgical anatomy and the more rapid identification and puncturing of the target calyx. The operative time is most likely to reflect a surgeon’s ability to perform the percutaneous surgery, and will include the time taken to reposition the patient after the placement of the ureteral catheter and to break up the stone, and a decline in the operative time can most likely be attributed to a surgeon’s familiarity with the instrumentation and improved dexterity. All of these parameters suggested that competence in US-guided PCNL can be achieved after 60 procedures. Our results concur with the findings from other studies. Ziaee et al[9] stated that a novice surgeon could gain competence in the procedure after completing 45 procedures. Allen et al[3] stated that a surgeon gained competence after 60 PCNL procedures, and that surgeons became excellent at the procedure after completing 115 procedures. In this study, a slight increase in the durations of the operations was observed between the 106^th^ and 120^th^ patients. This slight increase might have been associated with the surgeon’s interest in treating more complex stones and ensuring the patient was stone free after acquiring basic competence in the procedure.

Stone clearance is a very important clinical indicator of competency. In a study by Tanriverdi [6],the stone-clearance rate did not show any significant changes in sequential groups of patients. The authors concluded that a surgeon can meet this goal very soon after completing the first few procedures. We found high stone-free and CIRF rates for the novice surgeon that reached almost 95%, which suggests that the complete removal of all stones can be reliably achieved while the surgeon is learning the procedure. However, to achieve complete stone clearance, secondary treatments were sometimes needed. Although the stone-free rate increased with more practice, the difference was not significant.

In addition to treatment success, the numbers and severity of complications can be used as good indicators of competence. The Clavien-Dindo classification of surgical complications is a useful tool that can be used to objectively group adverse events according to their severity in PCNL[12]. In our patient series, major complications of Clavien-Dindo grades III–V did not occur. Neighboring organ injury and pneumothorax incidence rates were both 0%, and these might serve as excellent indicators of the low morbidity associated with US-guided PCNL. Performing the puncture under US guidance allows three-dimensional views of the kidney and the collecting system. Furthermore, neighboring organs such as the colon, spleen, and the lungs can easily be identified sonographically. Therefore, in contrast to performing punctures under fluoroscopic guidance, performing punctures under US guidance can safely avoid injury of the adjacent organs, which is of great importance to novice surgeons. However, the puncture technique requires a certain amount of training in US. Although not statistically significant, there was a trend towards fewer complications as the novice surgeon’s experience increased. The procedures performed on the 106^th^–120^th^ patients had increased transfusion rates, which were not statistically significant, and complication rates, which may have been associated with a higher percentage of larger stones. Given the reduction in the operative duration and the absence of complications as measures of competency, a novice surgeon would be able to reach these goals after 60 operations, and the competency level of the novice surgeon would be comparable with that of a senior surgeon. Traditionally, US guidance has been considered less accurate than fluoroscopic guidance; however, by using the two-step puncture technique described in our previous paper[11], accurate punctures can be achieved without difficulty during the early part of the learning curve.

The novice surgeon faced some difficulties during the first 60 procedures. First, the area surrounding the kidney became slightly swollen during surgery and, sometimes, this prevented a good view of the kidney. This was particularly apparent if the operative time was longer than 90 min. However, in most cases, the view was sufficient to permit checks for residual stones. Second, some residual stones migrated to the ureter during the operation, which led to misdiagnoses. Third, blood clots became positioned over the residual stones, which resulted in the inability to recognize the stones via nephroscopy and US.

This study has some limitations that should be considered. First, this study describes the PCNL learning curve for one surgeon who had experience in performing other endourologic procedures and who had observed 20 cases that included five cases during which he was the first assistant, before he undertook the procedure himself. Hence, he had extensive experience and his learning curve may not be applicable to someone emerging from their residency or someone who has little US training. Second, the data were collected retrospectively, but it is unlikely that a randomized prospective study with a similar design will be carried out in the future because patients will probably be unwilling to be randomized to a novice surgeon.

## Conclusion

US-guided PCNL can be performed safely and efficiently during the learning curve. Competence in PCNL is achieved after 60 procedures.
